# Uptake and trans-membrane transport of petroleum hydrocarbons by microorganisms

**DOI:** 10.1080/13102818.2014.906136

**Published:** 2014-07-02

**Authors:** Fei Hua, Hong Qi Wang

**Affiliations:** ^a^ Institute of Water Ecology and Environment, College of Water Sciences, Beijing Normal University, Beijing, P.R. China

**Keywords:** hydrocarbons, microorganisms, uptake, trans-membrane transport, outer membrane protein

## Abstract

Petroleum-based products are a primary energy source in the industry and daily life. During the exploration, processing, transport and storage of petroleum and petroleum products, water or soil pollution occurs regularly. Biodegradation of the hydrocarbon pollutants by indigenous microorganisms is one of the primary mechanisms of removal of petroleum compounds from the environment. However, the physical contact between microorganisms and hydrophobic hydrocarbons limits the biodegradation rate. This paper presents an updated review of the petroleum hydrocarbon uptake and transport across the outer membrane of microorganisms with the help of outer membrane proteins.

## Introduction

**Figure 1.**
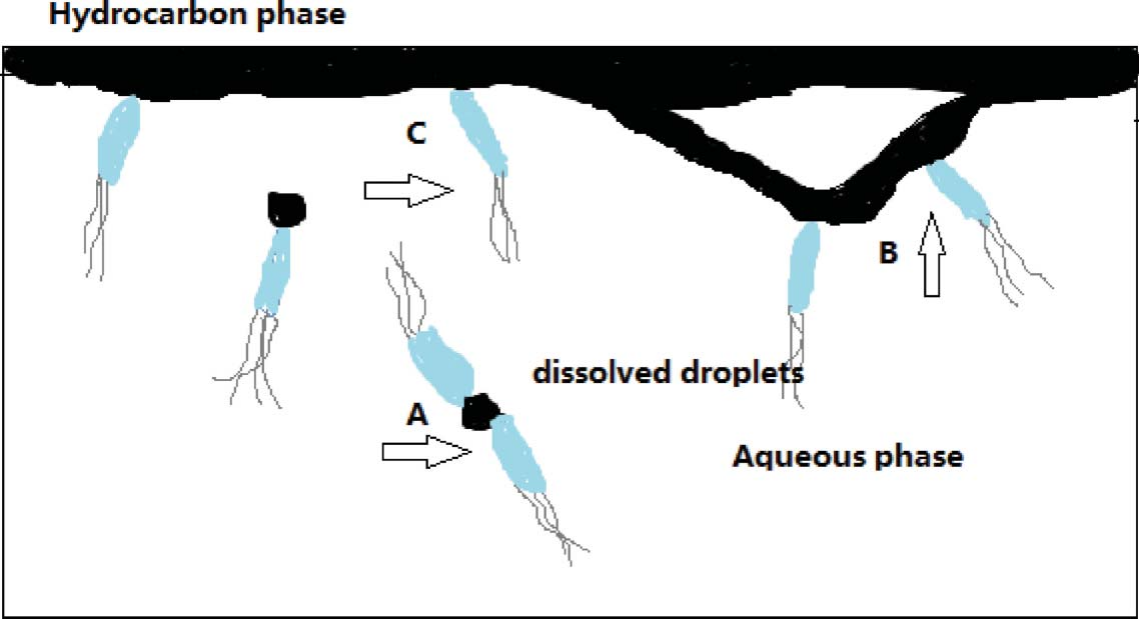
Three modes of hydrocarbon uptake by microorganisms with a petroleum oil phase floating on the aqueous phase.

Bioremediation of petroleum-contaminated soil is considered to be a very cost-effective technique, which mainly relies on the microorganism that can degrade hydrocarbons in the soil, such as bacteria and fungi.[1] These microorganisms are ubiquitous in nature and are able to utilize different hydrocarbons like short-chain, long-chain and aromatic hydrocarbons, including polycyclic ones, as a source of energy and carbon.[2,3] The membrane of bacterial cells is hydrophobic, which increases the difficulty of biodegradation for the decreased availability of hydrocarbons for uptake by bacterial cells. Thus, most substrates that can promote microbial growth need to undergo cellular attachment to become accessible by the cellular catabolic machinery. One of the important factors that limit biodegradation of oil pollutants is their limited availability to microorganisms,[4] and cell contact with hydrophobic compounds is a requirement before introduction of molecular oxygen into molecules by the cell-associated oxygenases.[5] The intracellular localization of hydrocarbon-degrading enzymes implies that there are three steps in the degradation of hydrocarbons by microorganisms. First, bacteria need to have access to the target compounds, i.e. the compounds have to be dissolved in the aqueous phase or bacteria have to directly adhere to the hydrocarbons; second, hydrocarbons that are adsorbed on the surface of the cell are transported across the membrane into the interior of the microorganism; finally, these hydrocarbons are degraded in the presence of enzymes, which is a quick process.[6] For Gram-negative or Gram-positive microorganisms, as shown in Figure 1, there are three different possible pathways for hydrocarbon uptake: (1) uptake of hydrocarbons dissolved in the aqueous phase; (2) contact of the cells with submicron oil droplets, i.e. the accommodated oil droplets; (3) direct contact of the cells with large oil drops.[7]

After the physical interaction between bacterial cells and hydrocarbons, the next step is trans-membrane transport of these hydrocarbons. Microbial cells have resources to physically access soluble, emulsified hydrocarbons and large oil droplets, transport these substrates across cell membranes,[8,9] and form inclusions before the hydrocarbons are metabolized.[10,11] Previous studies have reported on the trans-membrane transport of phenanthrene,[12] naphthalene [13] and *n*-hexadecane.[14] Substrate transport across the cell membrane can follow three main mechanisms: (1) passive diffusion; (2) passive facilitated diffusion; or (3) energy-dependent/active uptake. Metabolic inhibitors, such as sodium azide,[13] 2,4-dinitrophenol,[12] and carbonyl cyanide *m*-chlorophenylhydrazone (CCCP),[15] are often used to study if the transport of alkanes into microbial cells is through passive diffusion or through energy-dependent/active transport. Because the solubility of hydrocarbons, even alkanes, in the water phase is so low that analysing the content by standard methods like gas chromatography–mass spectrometry is not useful, isotope-labelling (e.g. with ^14^C) of hydrocarbons, like hexadecane, phenanthrene and naphthalene, is used to study the kinetics and specificity of the trans-membrane transport of hydrocarbons. On the other hand, recent studies have reported outer membrane (OM) proteins of Gram-negative bacteria involved in the transport of hydrocarbons across the cell membrane. The molecule structure of OM protein, including fatty acid degradation L (FadL) from *Escherichia coli*,[16,17] outer membrane protein W (OmpW) from *E. coli*,[18] toluene dioxygenation X (TodX) from *Pseudomonas putida* and toluene m-monooxygenation X (TbuX) from *Ralstonia pickettii*,[19] have been analysed. The results inferred that these OM proteins can facilitate the diffusion of small-molecule hydrocarbons into the cells of Gram-negative microorganisms. Different microorganisms might have different OM proteins involved in hydrocarbon transport across the cell membrane. Until now, OM proteins of Gram-positive microorganisms have not been reported. Meanwhile, OM proteins involved in energy-dependent transport have been poorly studied.

The uptake of hydrocarbons has thus been the subject of many studies, the majority of which focus on biosurfactants. However, the transport mechanisms by which hydrocarbons cross the cell membrane have received comparatively little attention, especially alkanes. This paper attempts to give an updated review on the uptake, trans-membrane transport mechanisms of petroleum contaminants as well as the OM protein involved in the transport process.

## Hydrocarbon uptake by microorganisms

### Uptake of hydrocarbons dissolved in aqueous phase

The first way of utilization is only suitable for water-soluble aromatics and short-chain hydrocarbons, but not for long-chain hydrocarbons or other high molecular weight substrates for their low solubility (10^−10^–10^−5^ g/L).[20] Generally speaking, microorganisms can grow well when their growth rate is slower than the dissolution rate of hydrocarbons, for there will always be soluble substrates in the environment. However, if the growth rate of the microorganism is quicker than the dissolution rate of hydrocarbons, then the biodegradation potential of the microorganism is limited. For example, some bacteria could grow well when naphthalene and 4-chlorinated biphenyl were served as the carbon source, as long as the biomass was small. However, when the concentration of hydrocarbons in the water could not be detected, the growth rate of bacteria dropped sharply.[21] Similarly, the degradation rate of phenanthrene by *Pseudomonas* was obviously limited to the dissolution rate of the substrate.[22] Studies have shown that exclusive direct interfacial uptake was utilized by 47% of studied strains that were isolated from the same petroleum contaminated soils, and a large proportion of strains (53%) produced biosurfactants, which suggested the existence of two distinct alkane uptake mechanisms in this group.[8]

### Uptake of pseudosolubilized hydrocarbon droplets

**Figure 2.**
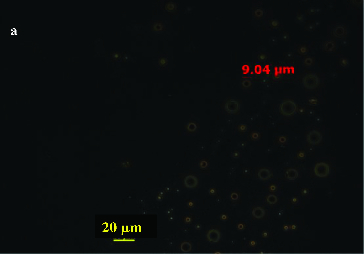
Phase-contrast micrograph of oil droplets in the culture medium (crude oil droplets in the water phase after incubation for 35 days – 40 magnification). Bar represents 20 μm. Reprinted with permission from Hua and Wang [32].

The uptake of pseudosolubilized hydrocarbons in a droplet form is very common and frequently involves the production of biological surfactant molecules (biosurfactant) as emulsifying agents to produce micro-droplets of hydrocarbons. Like all surface-active species, biosurfactants contain one or several lipophilic and hydrophilic moieties. The lipophilic moiety can be a protein or a peptide with a high proportion of hydrophobic side chains, but is usually the hydrocarbon chain of a fatty acid with 10–18 carbon atoms, although higher molecular weight fatty acids have been reported. The hydrophilic moiety can be an ester, a hydroxyl, a phosphate or carboxylate group or a carbohydrate.[23] Because of the amphiphilic property, many studies have shown that biosurfactants can emulsify hydrocarbons, thus enhancing their water solubility, decreasing surface tension, increasing the displacement of oily substances from soil particles, assisting the transport and translocation of the insoluble substrates across cell membranes, and helping detach the bacteria from the oil droplets after the utilizable hydrocarbon has been depleted.[24–27] On the other hand, in the case of water-insoluble substrates like *n*-alkanes and polycyclic aromatic hydrocarbon (PAH), the hydrophobic nature of the bacterial cell surface has been reported to play an important role, too. For example, *Mycobacterium* sp. LB501T exhibited a high specific affinity for anthracene and grew as a confluent biofilm on solid anthracene present as a sole carbon source, while no biofilm formation was observed on anthracene when excess glucose was provided as an additional substrate, since anthracene-grown cells were significantly more hydrophobic and more negatively charged than glucose-grown cells.[28] One group, the rhamnolipids, consisting of hydrophilic carbohydrates and long-chain aliphatic acids or hydroxy-aliphatic acids, are the most common class of the microbially produced surface active compounds and possess strong surfactant [29] and antibacterial and antiviral activities.[30] For example, *Pseudomonas* sp. DG17 could produce monorhamnolipids and dirhamnolipids when growing on octadecane.[31] And observation results from phase-contrast micrograph directly showed that crude oil components entered the water phase because of the pseudosolubilization effect of *Pseudomonas* sp. DG17, which indicates that the isolate could uptake pseudosolubilized crude oil droplets (Figure 2). Meanwhile, octadecane droplets in the culture medium were also observed by phase-contrast microscopy. The role of the biosurfactant is to increase the chance of direct contact between bacteria and oil droplets.[32] Furthermore, lipopeptides are another kind of biosurfactant produced by microorganisms. For example, ornithine lipids and the subtilysin produced by *Bacillus subtilis*, a Gram-positive bacterium, are claimed to be the most effective biosurfactant reported to date.[33] Studies have shown that this kind of biosurfactant has negative or positive effects on the biodegradation of hydrocarbons.[34]

Studies of Klein et al. [35] have shown that after having reached the hexadecane–water interface, by a purely thermal diffusion process, cells released surface-active compounds resulting in the formation of an interfacial viscoelastic film. The initial interaction occurred on metabolizable as well as non-metabolizable alkanes, indicating that at this stage cells are not affected by the nature of the alkane forming the interface. In contrast, at a later stage, the nature of the interface turned out to exert control over the behaviour of the cell.[35] By using ultrathin-section transmission electron microscopy, Southam et al. [36] observed that the assimilation of oil may occur via the fusion of tens of nanometer-sized droplets of emulsified oil with the OM of Gram-negative bacteria or through the uptake of surfactant complex oil. The extremely small droplets of emulsified oil at the bacterial cell surfaces and the extension of shiny organic material occurred between the bacterial surface and the surfactant boundary. Thus, incorporation of biosurfactant into the culture medium stimulated the degradation of these water-insoluble aromatic hydrocarbons.[37,38] Similarly, cells of *Pseudomonas* species grown on hydrocarbon were found to be connected to each other by means of numerous fibre-like projections and were found to be concentrated at areas of network formed by the extracellular secretion. The fibre-like network attracting bacterial cells observed in scanning electron microscopy could be a form of alkane and surfactant complex.[39] In addition, cell hydrophobicity is suggested to be a significant factor in microbial adhesion on surfaces including hydrophobic substrates.[40,41] The increase in cell hydrophobicity can be induced in the presence of biosurfactant combined with a slightly soluble substrate. Increased cell hydrophobicity promoted attachment of cells to hydrocarbon droplets, thus enhancing alkane degradation.[42] Chen et al. [43] found that in the presence of rhamnolipid, the microorganism adherence to hydrocarbon (MATH) of *Bacillus* sp. DQ02 increased 44% and the degradation of *n*-hexadecane increased 11.6%. Al-Tahhan et al. [4] showed that rhamnolipids caused an overall loss in cellular fatty acid content. The amount of lipopolysaccharide (LPS) loss was found to be dependent on rhamnolipid concentration, but significant loss occurred even at concentrations less than the critical micelle concentration. The authors conclude that rhamnolipid-induced LPS release is the probable mechanism of enhanced cell surface hydrophobicity.[44] Nikaido [45] suggested earlier that lost LPS may be replaced with phospholipids, resulting in a cell surface with increased hydrophobic character. Although the possible incorporation of surfactants into the cell membranes may contribute to the changes in the cell surface properties, a drastic decrease of the cell surface hydrophobicity may occur, which in turn may cause a negative effect, considering highly hydrophobic species, using a substrate uptake mechanism through direct contact.[46]

Bioavailability and biodegradation kinetics of the hydrophobic pollutants are affected variably by the surfactants.[47] Either stimulating or inhibiting effects of surfactants on bioremediation of pollutants are known depending on the chemical characteristics of the surfactant, pollutant and physiology of the microorganism.[48,49] Some studies demonstrate that the biosurfactant produced by a microorganism sometimes could not initiate the degradation of hydrocarbons.[50,51] Bouchez-Naitali and Vandecasteele [8] showed that biosurfactants produced by strain *Rhodococcus equi* Ou2 had only a minor role in hexadecane degradation, for the major mode of alkane uptake was likely to be direct contact with large oil droplets. On the contrary, biosurfactants produced by *Pseudomonas aeruginosa* GL1 could stimulate hexadecane degradation, and their pseudosolubilization capacity, rather than their emulsification capacity, was involved in substrate degradation. At a surfactant concentration significantly below the critical micellar concentration (CMC) value, no enhancement or inhibitory effect on biodegradation is observed, whereas at or above the CMC value biodegradation is inhibited, suggesting that the substrate contained within the micelles is not bioavailable.[47] For example, Sotirova et al. [52] have shown that the presence of low concentration of biosurfactant (rhamnolipid + alginate) in the culture media was neutral to the growth of Gram-positive *B. subtilis* and Gram-negative *P. aeruginosa*, but only detrimental to *B. subtilis* when the concentration of biosurfactant was greater than CMC. On the other hand, added rhamnolipids above CMC have been shown to enhance the apparent aqueous solubility of hexadecane and the biodegradation of hexadecane, octadecane, *n*-paraffins, creosotes and other hydrocarbon mixtures in soil, as well as to promote the bioremediation of petroleum sludges.[15,53] This could be explained with the fact that above the CMC, formation of micelles occurs, and hydrocarbons can partition into the hydrophobic micellar core, increasing their apparent aqueous solubility. These opposite effects appear to be related to the composition of the bacterial cell surfaces. It is well known that Gram-negative bacteria, unlike Gram-positive ones, have a unique OM containing LPS, porin channels and murein lipoprotein.[54] Increased cell permeability induced by biosurfactant PS is most likely caused by the release of LPS from the OM.[44]

### Direct contact with large hydrocarbon drops

For this hydrocarbon uptake way, microbial cells directly attach to the liquid hydrocarbon drops that are much larger than the cells. The microorganisms that grow in the fatty acids not in the water phase often combine with hydrocarbons to form an agglomeration that can bring the microorganism cells in close contact with the hydrocarbons. Different microorganisms have different affinity to hydrocarbons. Goswami and Singh [55] found a *Pseudomonas* strain that could adhere to the surface of hexadecane and directly transport the substrate into the cells without secreting extracellular surfactant or emulsifier. What is more, Coimbra et al. [56] found that yeast cells showed high or intermediate hydrophobicity and interfacial tension values and low values of surface tension, which indicated that the cells utilize two mechanisms for uptake of insoluble substrate, i.e. direct interfacial uptake and biosurfactant-mediated transfer. Furthermore, sorption of phenanthrene to humic acids (HA) aggregates created a barrier to bioavailability that was breached only by a specific group of competent bacteria which were capable of interacting with HA in such a way as to gain access to the HA-sorbed phenanthrene, supplementing diffusive uptake from the freely dissolved phase.[57,58] Pini et al. [59] found that isolates assigned to the genus *Rhodococcus* produced a biosurfactant, whereas *Alcaligenes* strains modified their cellular envelope rendering it hydrophobic to react with hydrocarbon molecules. When the two strains were grown together in the presence of diesel fuel as the sole carbon and energy source, a positive and beneficial interrelationship between them occurred, suggesting that a sort of ‘functional complementation’ between the two strains might exist. Thus, different microorganisms can work together through different strategies to access to hydrocarbons.

Biosurfactants produced by *Pantoea* species A-13, when grown on kerosene or *n*-paraffins, increased the cell hydrophobicity and enhanced both the surface-tension–lowering capacity and the emulsifying potential. This suggested the occurrence of both modes of biosurfactant-enhanced growth on tested hydrocarbons: (1) direct contact with large alkane droplets and (2) alkane transfer mechanism which involves solubilization and emulsification of hydrocarbons at higher concentrations of biosurfactants in the culture medium.[60] Similarly, direct contact with big oil droplets and emulsified droplets simultaneously was also reported by Hua and Wang.[31]

**Figure 3.**
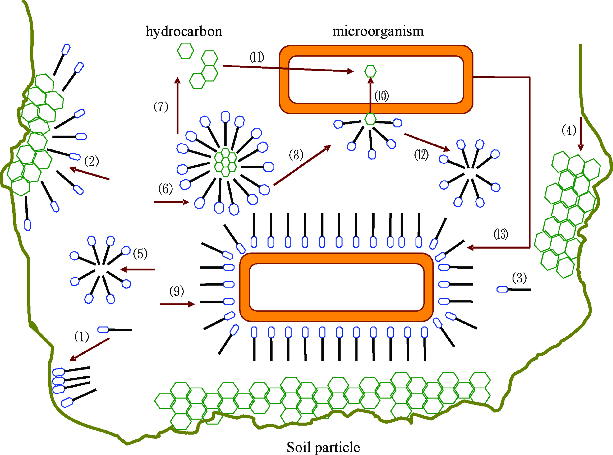
Uptake of hydrophobic hydrocarbon by microorganisms.

Many studies have reported the uptake of emulsified small oil droplets with the incorporation of biosurfactant.[53,61,62] The uptake mechanism linked to the attachment of cells to an oil droplet is still unknown but production of biosurfactants has been well studied. Microorganisms have evolved different natural methods of uptake of hydrophobic compounds, in general reflected by their capacity to generate hydrophobic cell surfaces or to secrete biosurfactants into the surrounding medium.[47] The efficacy of added surfactants will relate to the extent that they enhance, antagonize or repress the natural capabilities of the microbes in accessing the contaminants. The beneficial or negative impacts of added surfactants will be more clear-cut in pure culture systems than in indigenous mixed populations where only the aggregate of positive and negative effects will be observed.[47] Figure 3 shows the uptake ways of hydrocarbons in the environment: (1) biosurfactant that is adsorbed on the soil particle; (2) biosurfactant that is adsorbed on the hydrocarbon; (3) biosurfactant molecule that is dissolved in the water phase; (4) hydrocarbon that is adsorbed on the soil particle; (5) biosurfactant micelle; (6) hydrocarbon that is pseudosolubilized in the biosurfactant micelle; (7) hydrocarbon that migrates from the organic phase to the water phase; (8) micelle that is attached to the cell surface of the membrane; (9) biosurfactant attached to the surface of the cell membrane; (10) uptake of pseudosolubilized hydrocarbon droplets in the micelle; (11) uptake of hydrocarbon droplets dissolved in the water; (12) micelle that is detached from the cell membrane; (13) microorganism that is adsorbed on the surface of a hydrocarbon drop under the effect of biosurfactant and (14) intracellular inclusions.

## Trans-membrane transport of hydrocarbons

### Passive diffusion of hydrocarbons

Kallimanis et al. [12] suggested that phenanthrene uptake in glucose-grown cells was very slow and exhibited linear, non-saturable kinetics. On the contrary, cells grown on phenanthrene exhibited high initial uptake rates and followed Michaelis–Menten kinetics with high affinity for phenanthrene uptake. The ^14^C phenanthrene uptake reached a steady-state level at 15 min, which was 3.12 nmol/mg dry weight of cells, about 26 times higher than that of the glucose-grown cells. Similarly, kinetic analysis revealed that linear partitioning is not the only mechanism that explains the immediate uptake in induced cells and suggests that phenanthrene is specifically bound to induced cells, based on the saturation kinetics and entered into the cells by passive diffusion.[63] Verdin et al. [64] first demonstrated that simultaneous addition of sodium azide, an inhibitor of the electron-flow chain in oxidative phosphorylation, and benzo[a]pyrene to a six-day-old *Fusarium solani* culture, and addition of benzo[a]pyrene only to a 48-hour-old culture of *F. solani* did not affect the benzo[a]pyrene uptake and storage, which indicated that benzo[a]pyrene incorporation was not ATP dependent. Naphthalene can be transferred smoothly to the bacterial cells of *P. putida* through simple diffusion even when ATP inhibitors have been added in the extracellular matrix.[65] Moreover, phenol at higher concentrations can enter the cells of degrading bacteria by passive transport, but when the concentration is less than 50 mg/L, bacteria transfer phenol into the cells through active transport.[66] Naphthalene transport in cells of *P. putida* was not coupled to an energy source and was not inhibited by the presence of azide, suggesting that a diffusion process took place; however, this conclusion needs to be further tested according to the different concentration of inhibitors.[13]

### Energy-dependent active transport of hydrocarbons

Some hydrocarbons can be biodegraded in the external medium under the presence of extracellular oxygenases, but others travel across bacterial membranes to reach the cytoplasmic metabolic enzymes for intracellular degradation. Studies have shown that many hydrocarbons that are adsorbed on the surface of microbial cells can be transported across the membrane into the cells mainly through passive transport or active transport. Organic compounds such as PAHs, due to their low water solubility and high octanol-water partition coefficients, tend to partition into cell wall structures. Such movement is generally brought about by passive transport down a concentration gradient from the environment into the cell.[67] To study whether the transport of hydrocarbons is induced by the substrate or not, the uptake of ^14^C-labelled hydrocarbons and non-labelled hydrocarbons, like hexadecane, naphthalene, phenanthrene, were studied at the same time. Kallimanis et al. [12] showed that sodium azide, or 2,4-dinitrophenol, an uncoupler of oxidative phosphorylation, dramatically reduced the uptake of phenanthrene in phenanthrene-grown cells, which showed that phenanthrene travels across the bacterial membrane via a phenanthrene-inducible active transport mechanism that is dependent on the proton motive force. On the other hand, some studies infer that the transport of substrate is also related with the substrate concentration in the environment. It is evident that diverse adaptations for efficient transport of PAHs have evolved in PAH-utilizing bacteria and different transport processes may play a role in the mineralization of low concentrations of cyclic hydrocarbons.[68] Recently, Hua et al. [69] found that adding sodium azide at the beginning of cultivation also decreased the intracellular ^14^C-labelled octadecane of *Pseudomonas* sp. DG17, which demonstrated that *n*-hexadecane could be transported across the bacterial membrane in an energy-dependent manner. It was also found that octadecane entered the cell through active trans-membrane transport when the substrate concentration is low, while a diffusion process was observed at high substrate concentrations. Moreover, the entry of phenanthrene into acetate-grown cells of *Mycobacterium* sp. strain RJGII-135 is a non-saturable, linear partitioning process.[63] Similar active transport systems have also been described for naphthalene uptake by gamma-proteobacterium *Pseudomonas fluorescens* Uper-1.[13] Take another cytochrome oxidase inhibitor, CCCP as an example, the larger the dose of CCCP, the larger the decrease in hexadecane uptake; which indicates that some form of energy-dependent mechanism mediates the process of transport into bacterial cells, and blocking all energy-dependent cell mechanisms also caused biosurfactant production to cease.[15] Obviously, cytochrome oxidase inhibitors blocking the proton motive force of electron transport phosphorylation has a deeper relationship with the uptake and trans-membrane transport of hydrocarbons, which needs further study.

On the other hand, studies on the trans-membrane transport of PAHs or alkanes also inferred that induced cells and uninduced cells utilized different transport ways for uptake of extracellular substrate. For uninduced cells, steady-state concentrations of cellular phenanthrene were achieved within the initial 15 s of incubation. The lack of saturation and the lack of inhibition by azide and CCCP support the finding that this immediate uptake is caused by passive diffusion.[63] However, the induced cells showed a cumulative uptake curve over a period of a few minutes when incubated with ^14^C phenanthrene. Since phenanthrene enters immediately the uninduced cells by passive diffusion, the continued cumulative uptake in induced cells must also involve a constant role of partitioning. Kinetic analysis revealed that linear partitioning is not the only mechanism that explains the immediate uptake in induced cells and suggests that phenanthrene is specifically bound to induced cells, based on the saturation kinetics.[15,63] Moreover, if naphthalene was transported in the cell, the level of intracellular ATP would be expected to be reduced as naphthalene is metabolized during the incubation. This was not observed during the incubation, since the level of intracellular ATP did not decrease in the azide-treated cells. Therefore, the inhibition of naphthalene uptake cannot be due to a general effect on metabolism, but instead due to the loss of the cell's ability to transport naphthalene, and the concentrations of inhibitors should be selected carefully in order to inhibit cell growth completely.[13]

### Formation of intracellular lipid inclusions

**Figure 4:**
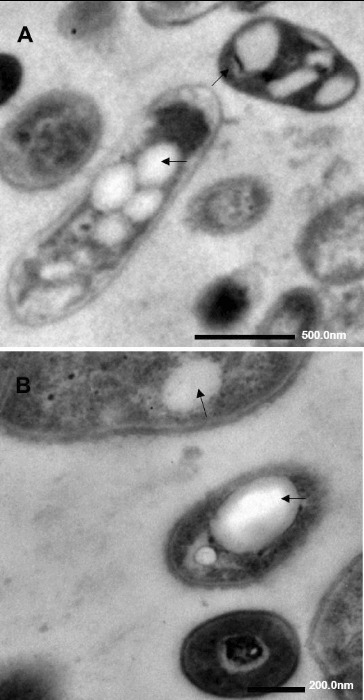
Transmission electron microscopy of DG17 grown on 400 mg/L *n*-octadecane. Arrows show inclusions of *n*-octadecane. Reprinted with permission from Hua et al. [31].

Petroleum, as a substrate, usually could be incorporated into the cells by a specific and inducible transport system, which is particularly important for oil biodegradation. Accumulation of storage lipids is one of the strategies microorganisms have developed to facilitate their survival in variable environments.[11,70] For example, Alvarez et al. [71] found various types of intracytoplasmic sudanophilic, electron-transparent inclusions and electron-dense inclusions in the cells of *Rhodococcus opacus* strain PD630 during cultivation on gluconate and phenyldecane. These inclusions occurred predominantly a sphere-shaped structure, and disc-shaped inclusions which exhibited at a much lower number both in the gluconate-grown and phenyldecane-grown cells. Similarly, it was found that *Pseudomonas* sp. DG17 grown on *n*-octadecane displayed rounded clear-vesicle type inclusions (Figure 4).[69]

The composition of inclusions, which indicated a function of the inclusion bodies as a carbon and energy store, such as lipids and lipophilic compounds, is different. In the cells of *R. opacus* strain PD630, when phenyldecane served as the carbon source, the lipids in the inclusions consisted predominantly of saturated and unsaturated straight long-chain fatty acids (LCFAs); however, 3-hydroxyalkanoic acids or other hydroxyalkanoic acids were not detected under any cultivation conditions.[71] Alvarez et al. [10] found that many psychrophiles could accumulate specialized lipids like poly-hydroxyalkanoic acids (PHA), and the compositions of the accumulated polyesters were mainly medium-chain-length hydroxyalkanoic acids like 3-hydroxybutyric acid, 3-hydroxyvaleric acid, 3-hydroxyhexanoic acid, with 3-hydroxydodecanoate as the predominant constituent of the polyester. Deandres et al. [11] found that the major lipid classes in the extracts from cells of *P. aeruginosa* 44T1 cultivated on olive oil, glucose or *n*-alkanes were diacylglycerols and triacylglycerols, and a relatively high concentration of linolenic acid and arachidonic acid was detected when the strain was incubated in glucose and *n*-alkane medium. Barabas et al. [72] analysed the constituent fatty acids of total lipids from a C_16_ utilizer *Streptomyces* strain and the results showed an increased proportion of *n*-hexadecanoic acid, which suggested that the alkane-utilizing microorganisms tended to accumulate fatty acids with chains equivalent in length to those of the alkane substrate. Thus, it is known that hydrocarbons are taken up unaltered by microorganisms, and are subsequently oxidized intracellularly.[34,73]

## Outer membrane proteins involved in trans-membrane transport of hydrocarbons

Bacterial biodegradation of hydrocarbons, an important process for environmental remediation, requires the passage of hydrophobic substrates across the cell membrane.[19] Studies have shown that the oligosaccharide moieties of LPS in the OM outer leaflet can extend out from the membrane surface by as much as 30 Å, providing a very effective barrier to hydrophobic molecules. The most frequently studied exemplar of OM transport of hydrophobic molecules is FadL,[17,74,75] which is responsible for the uptake of LCFAs. These hydrophobic compounds can cross the cell membrane through diffusion without energy or in an energy-dependent (active) manner. With rare exceptions, virtually all OM proteins are β-barrels, consisting of an even number of 8 to 24 β-strands forming a pore-like structure. Many of these OM pore-like β-barrels are classified as porins, and most nutrient uptake is accomplished by them.[75]

Porins function passively, permitting the energy-independent diffusion of solute molecules with a molecular mass of 600 Da or less downhill across a concentration gradient, through the porin's β-barrel, and into the periplasm.[75,76] The LCFA transporter FadL from *E. coli* (EcFadL) is the archetypal member of a large family of OM proteins mediating the passive diffusion of hydrophobic molecules across the OM.[16,17,77] Moreover, recent studies indicated that high-affinity substrate binding to the OM LCFA transporter FadL from *E. coli* causes conformational changes in the N terminus that open up a channel for substrate diffusion. The OM LCFA transporter FadL from *E. coli* is a unique paradigm for OM diffusion-driven transport, in which ligand gating within a β-barrel membrane protein is a pre-requisite for channel formation.[77] The crystal structures of two OM proteins, *P. putida* TodX and *Ralstonia pickettii* TbuX, which have been implicated in aromatic hydrocarbon transport and are part of a subfamily of the FadL, were also reported.[26] The TodX and TbuX structures revealed 14-stranded β-barrels with an N-terminal hatch domain blocking the barrel interior. A hydrophobic channel with bound detergent molecules extends from the extracellular surface and is contiguous with a passageway through the hatch domain, lined by both hydrophobic and polar or charged residues. The TodX and TbuX structures support a mechanism for transport of hydrophobic substrates from the extracellular environment to the periplasm via a channel through the hatch domain.[19]

Another family of proteins, *E. coli* OmpW, could be involved in the transport of small hydrophobic molecules across the bacterial OM in Gram-negative bacteria. Studies have shown the crystal structure of *E. coli* OmpW to 2.7 Å resolution.[18] The structure shows that OmpW forms an eight-stranded β-barrel with a long and narrow hydrophobic channel that contains a bound *n*-dodecyl-N,N-dimethylamine-N-oxide detergent molecule. Single channel conductance experiments show that OmpW functions as an ion channel in planar lipid bilayers. The channel activity can be blocked by the addition of *n*-dodecyl-N,N-dimethylamine-N-oxide.[18]

Meanwhile, for Gram-negative bacteria, there are proteins that allow the transport of molecules too large or too scarce to accumulate by use of the TonB-dependent OM active transport system. These specialized, ligand-specific, active transport proteins move compounds against a concentration gradient across the OM through the proton-motive force of the inner membrane through physical interaction with TonB-ExbB-ExbD, an inner membrane complex.[78,79] In addition, the crystal structure of an active transport, OM receptor at 2.4 Å resolution was reported by Buchanan et al. [78]. Two distinct functional domains are revealed: (1) a 22-stranded β-barrel that spans the OM and contains large extracellular loops which appear to function in ligand binding and (2) a globular N-terminal domain that folds into the barrel pore, inhibiting access to the periplasm and contributing two additional loops for potential ligand binding. These loops could provide a signalling pathway between the processes of ligand recognition and TonB-mediated transport. The blockage of the pore suggests that the N-terminal domain must undergo a conformational rearrangement to allow ligand transport into the periplasm.[78]

## Conclusions

The uptake of hydrocarbons, especially of low-molecule-weight alkanes and PAHs, has long been known and many of the basic features of biosurfactant produced by degradative organisms have been well reported. Although direct contact of the cells with submicron oil droplets by producing biosurfactant is the major mechanism for microorganisms to attach hydrophobic petroleum compounds, uptake of dissolved droplets or direct contact with big oil droplets are also reported. Cell hydrophobicity is an important factor that could influence the physical contact between microorganisms and hydrocarbons. Increasing the cell membrane hydrophobicity could help microorganisms to directly make contact with big oil droplets. Moreover, biosurfactants can help to increase the cell membrane hydrophobicity of microorganisms. Further studies are still needed to elucidate whether two or more microbial uptake ways occur at different times when microorganisms utilize a specific hydrocarbon. Meanwhile, biosurfactants can inhibit the biodegradation of hydrocarbons which infers that the effect of biosurfactants produced by microorganisms needs insight studies. Further studies should focus on how the rhamnolipid molecules interact with the cell surface chemical groups in order to achieve quantitative control of the cell surface hydrophobicity by the surfactant and successful application of the technique in bioremediation of contaminated soils or other fields.

There are limited investigations on the other key step in bacterial biodegradation of hydrocarbons, how these hydrophobic hydrocarbons cross the cell membrane. Until now, in most studies, isotope was used to study the cellular hydrocarbons to analyse whether the trans-membrane transport process needs energy. However, there are few reports on the transport of high-molecular-weight hydrocarbons, such as C_20_–C_30_ alkanes. On the other hand, the detailed molecular processes involved in microbial trans-membrane transport of hydrocarbons also require further elucidation. For Gram-negative bacteria, OM proteins are reported to be involved in the transport of hydrocarbons across the cell membrane. The crystal structure of the *E. coli* long-chain fatty-acid transporter FadL, a member of a distinct and conserved family of OM proteins, and the OmpW family involved in the uptake of hydrophobic compounds, are most studied. The two kinds of OM proteins can help small hydrophobic molecules cross the bacterial OM through passive diffusion. However, active transport proteins are poorly studied, and only the TonB-dependent OM system has been found to be involved in the active transport process. In addition, the studies on the OM proteins involved in trans-membrane transport of hydrocarbons have been mainly concentrated on the Gram-negative microorganism *E. coli*. However, the membrane proteins involved in hydrocarbon transport of other microorganisms, including *Pseudomonas*, *Rhodococcus*, *Acinetobacter*, *Mycobacterium*, *Arthrobacter* and *Burkholderia*, are not clear.

Therefore, based on the current state of knowledge reviewed here, it may be concluded that the uptake and trans-membrane transport of hydrocarbons can be considered as the new key step in the remediation of petroleum hydrocarbons. 
